# A modelling approach to assess the impacts of climate dynamics and anthropogenic pressure on water yield in the Damodar River basin

**DOI:** 10.1038/s41598-025-29098-9

**Published:** 2025-11-23

**Authors:** Jyoti Prakash Hati, Anirban Mukhopadhyay, Rituparna Acharyya, Halina Kaczmarek

**Affiliations:** Faculty of Geographical Sciences, Kazimierz Wielki University, Bydgoszcz, Poland

**Keywords:** InVEST model, Annual water yield, Gray level co-occurrence matrix, Random forest, Damodar river, Climate sciences, Environmental sciences

## Abstract

Changes in climatic variables like precipitation and the rapid development of anthropogenic activities have the potential to affect both groundwater and surface water. It is specifically observable in the non-perennial river like the Damodar, where, despite the construction of dams, flooding is a recurring problem. This study aims to evaluate the changes in the annual water yield of the DRR using the ‘annual water yield’ module of the Integrated Valuation of Ecosystem Services and Tradeoffs (InVEST) model. The inputs of the model were derived from different remote sensing datasets using the Google Earth Engine (GEE) platform. The results indicate a lower annual water yield in 2023 (mean 1036 mm) and a higher yield in 2013 (mean 1426 mm), compared to 2003 (mean 1219 mm). Reduced vegetation cover (reduced by 5500 km^2^), reduced area of waterbodies (471 km^2^), and increased urbanisation (built-up areas increased by 700 km^2^) were observed between 2003 and 2023 in the DRR. The sensitivity analysis of the model revealed that precipitation is a more influential factor than evapotranspiration when simulating water yield. The effect of changing land use was found to have very little or no effect on the annual water yield. This study also demonstrates a coupled framework for building hydrological resilience in the DRR by integrating three subsystems – namely, the water subsystem, the human subsystem and the socio-hydrological system.

**Supplementary Information:**

The online version contains supplementary material available at 10.1038/s41598-025-29098-9.

## Introduction

In the modern era, the rapid expansion of industries, agriculture and other activities has significantly changed land cover throughout the world. Changing land cover has caused several problems, including soil degradation^1^, increased land surface temperature^2^, the formation of heat islands^3^, degraded ecosystem services^4^, degraded air quality^5^, etc. Temporal changes in land use patterns could have a significant impact on meteorological variables like precipitation and evapotranspiration, affecting both surface and groundwater systems^6^. Anthropogenic modifications to land use are also connected to sediment transport^7^, river ecosystem^8^ and water quality^9^. Therefore, the impact of land use on hydrology is crucial, as it affects the availability of water resources, which are vital for both humans and the ecosystem^10^. Land cover, soil and topography are primarily thought to be the most important parameters regulating rainfall-runoff dynamics^11^. Since soil and topography have very little immediate impact, changes in land use are considered to comprise the single most important factor in influencing rainfall-runoff processes^12^. As climatic effects and their consequences (like flooding) across the world are going to be exacerbated in future^13^, the impact of land use changes on hydrology is expected to gain much attention.

The effects of land use change on regional hydrology are often complex to evaluate, as climatic parameters also influence the process. The effects of land use on hydrology can be separated from climatic parameters using paired catchment studies, statistical models generated from water balance, and hydrological models. Although paired catchment studies eliminate climatic factors, they require a long calibration time and are only suitable for smaller basins^10^. Water balance equations, on the other hand, do not effectively distinguish land use and climatic effects. The modelling strategy is dependent on a dense network of automated rain and river gauging stations, which precludes its use in data-scarce regions such as the DRR. With limited data, the uncertainty in the parameters (like soil moisture and channel morphology) and structure of the hydrological model will be a problem, and it will be challenging to evaluate the findings and see how they accurately reflect reality^11^. This is one of the reasons behind the scarcity of modelling approaches to DRR in the literature.

Therefore, in this paper, the impact of land use change on the hydrology of the DRR was evaluated using geospatial techniques and the Integrated Valuation of Ecosystem Services and Trade-offs (InVEST) annual water yield model. The Water Yield model calculates the average yearly water generated by a watershed, and it can run in a data-scarce region^14^. On the other hand, geospatial techniques are adequate for quantifying the temporal changes in land use, and they can also provide estimations on related parameters like evapotranspiration, Leaf Area Index (LAI), precipitation, and impervious surface. Several studies have employed land use change over the DRR for different purposes, like flood mapping, water quality, etc^15,16^. Changes in hydrological conditions due to land use change are still an unexplored area in DRR. As the demand for freshwater is sharply increasing in this region due to multiple industries and a growing population, there is a serious need for a model for water resource management and regulations. Currently, Damodar Valley Corporation (DVC) allocated 616 million cubic meters (MCM) for Jharkhand state and 697 MCM for West Bengal during 2021–22^17^ for municipal, domestic and industrial requirements. This research aims to address the knowledge gap in evaluating the changes in the annual water yield of the tested basin by utilizing the potential of remote sensing datasets and modern, sophisticated algorithms. This study will serve as baseline information on decadal changes in land use and its impact on the main hydrological factors in the river basin. This study will provide insights to improve the resilience of the hydrological system in the Damodar watershed by implementing specific and targeted adaptation or mitigation strategies based on the resilience framework.

## Methods

### Study area

**Fig. 1:**
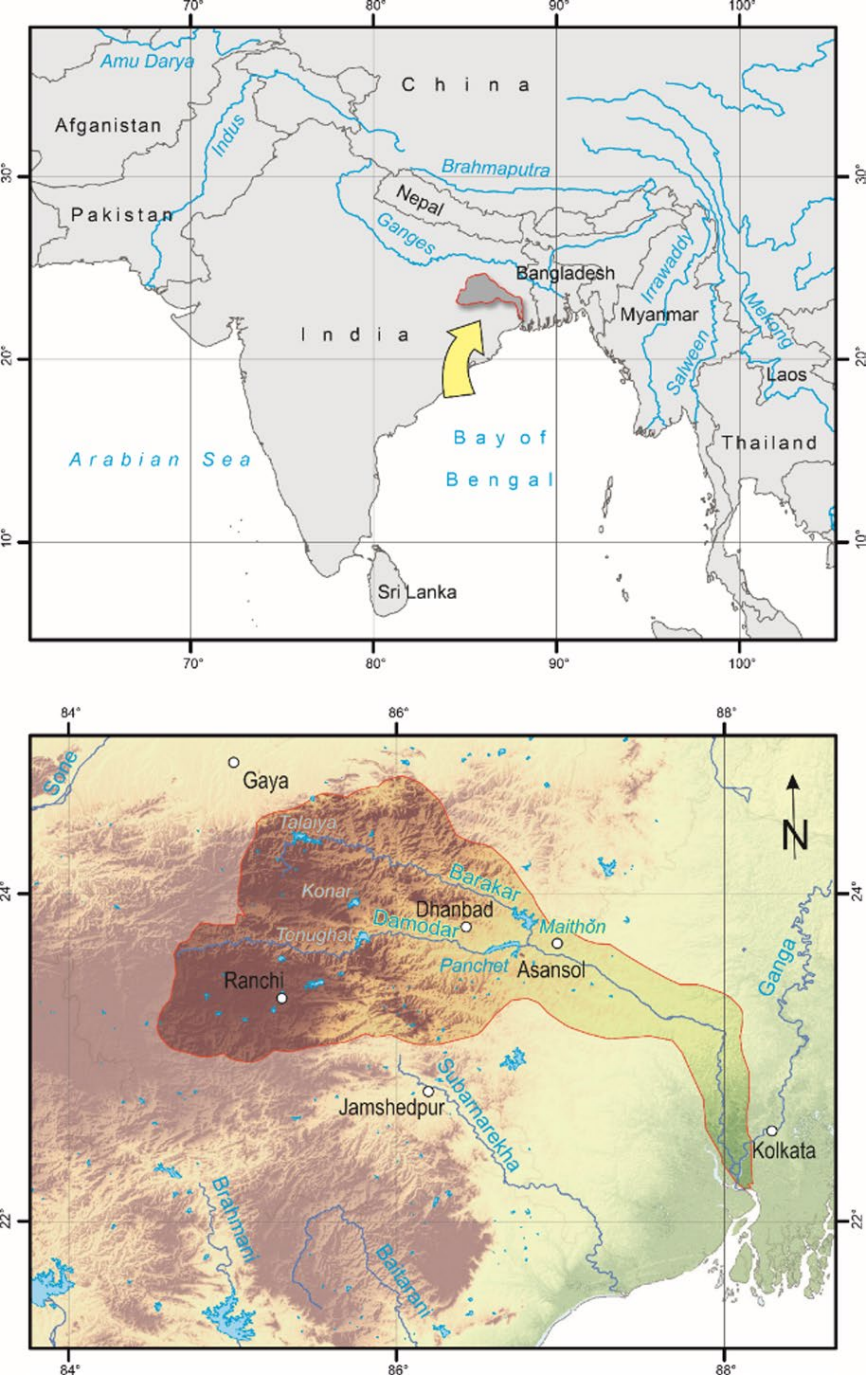
Study area, showing the Damodar River Region. Source: authors’ own work.

The Damodar River originates near the Palamau hills at Jharkhand and flows south-eastwards for 547 km to join the Hugli River, a distributary of the Ganges in West Bengal, India^18^ (Fig. 1). The upper catchment of the DRR in Jharkhand has mineral-rich hills, plateaus and sloping plains, whereas the lower catchment area in West Bengal has fertile land and high agricultural productivity^19^. The Damodar basin has 46% of India’s coal reserves and is called the “storehouse of Indian coal”^20^. In the upper section of the basin, coal mining and mine-based industrial activities contribute significantly to the economy, which has also rendered the valley prone to soil erosion. With the changing climate, an increase in streamflow is predicted in the future^21^, which will create flood-related problems because the dams have lost a significant amount of storage capacity due to sedimentation^22^.

The average height of the DRR ranges from 65 m in the lower catchment to 1,374 m near the origin^23^. In this study, along with the Damodar River, its tributaries were also considered, as they transport water to the five reservoirs (Tilaiya, Maithon, Konar, Tenughat and Panchet) (Fig. 5). So, the inputs for the model simulation were generated for a 40,553 km^2^ area. Daily temperature ranges from a low of 3 °C in winter to a maximum of 40 °C in summer^24^. The average yearly rainfall is 1,250 mm, but more than 80% of rainfall occurs in the monsoon^25^, which often leads to floods in lower catchments. The Damodar River earned the name “Sorrow of Bengal” due to frequent flooding in the past. In the 1950s, a coordinated two-stage development programme was put into place for the construction of several dams across the Damodar and its tributaries. The construction of dams at Tenughat and Panchet, as well as a barrage at Durgapur on the Damodar River, was done for irrigation, electricity production, flood control, and soil preservation. Later, three dams (Tilaiya, Maithon and Konar) were built on tributaries of the Barakar and Konar rivers^26^. However, because of heavy siltation, the flooding occurrence has not been solved. Massive amounts of silt are deposited in these reservoirs from the surface runoff of the upper catchment during the monsoon^27^, reducing their storage capacity. Water shortage, on the other hand, has emerged as a critical concern in certain regions where groundwater extraction is prevalent, particularly in agricultural areas of the upper catchment^23^. As a result, the water table is gradually declining in some areas of the river basin, forcing people to drill deeper^28^.

### Datasets used

Table 1 represents the datasets used in the present study. The precipitation and evapotranspiration data were processed in Google Earth Engine (GEE). Both datasets were clipped according to the study area boundary and exported from GEE in raster format. Similarly, root-restricting layer depth and plant-available water content data were also clipped from a globally available layer. Other datasets, such as land use, biophysical table, Z parameter and watershed boundary, were prepared by the authors. The model was simulated for the years 2003, 2013 and 2023 to observe whether a decadal pattern emerges from the results. The starting year is taken as 2003, as the MODIS Aqua launch date was May 2002; therefore, the evapotranspiration datasets for a full year were available in 2003.

**Table 1:** Details of the datasets used in the current research.

Parameters	Description	Source
Precipitation	CHIRPS Daily: Climate Hazards Center InfraRed Precipitation With Station Data (Version 2.0 Final)	Earth Engine Data Catalogue
Evapotranspiration	MODIS Terra Net Evapotranspiration	Earth Engine Data Catalogue
Root Restricting Layer Depth	Depth to bedrock or the depth below ground where root penetration is restricted	The Commonwealth Scientific and Industrial Research Organisation (CSIRO)
Plant Available Water Content	Describe the amount of water in the soil available to plants	^29^
Land Use/Land Cover	Analysed from Landsat TM (for 2003) and OLI (for 2013 and 2023) datasets	Authors
Biophysical Table	A table containing biophysical parameters for all the LULC classes	Authors
Z Parameter	A constant, typically calculated as 0.2×N where N is the number of rain events in a year	Authors
Watersheds	The course was determined based on the TanDEM-X 30 m digital elevation model using the Flow accumulation tool in SAGA GIS	Authors

## Methodology

### Model description

Below, we present the results of the use of modelling tools in assessing the impact on the hydroecosystem, as implemented as part of the Integrated Valuation of Ecosystem Services and Trade-offs (InVEST) model developed by scientists from Stanford University, University of Minnesota, the Chinese Academy of Sciences, The Nature Conservancy, the World Wildlife Fund, the Stockholm Resilience Centre and the Royal Swedish Academy of Sciences^30^. The InVEST model is a continually developed software package that quantifies and maps the values of ecosystems. This widely used^14^ model is divided into 19 core ecosystem modules, including an annual water yield module and four support tools. The InVEST model is currently deployed in various ecosystem mapping and monitoring GIS applications, and preliminary calibration experiments emphasise the model’s sensitivity to certain input factors^31^. The InVEST Water Yield model provides information on how variations in land use patterns impact the yearly surface water yield by estimating the proportional contributions of water from various landscape features. The InVEST workbench version 3.14.2 was simulated for estimating annual water yield in the DRR. The annual water yield model has been used by several scientists around the world^32–34^. Initially, the model calculates the quantity of water evaporating off each pixel by subtracting the percentage of water that evapotranspires from the precipitation. The model assumes that all water supply from a pixel reaches the place of interest via either surface or subsurface baseflow. The water yield is then averaged and summed up to the sub-watershed level in this model. The user may depict the heterogeneity of important parameters influencing water yield, such as plant type, precipitation, soil type, etc., using pixel-scale computations.

The Budyko curve and yearly average precipitation serve as the foundation for the water yield model. For every pixel in the landscape, the annual water yield is calculated using Eq. (1) as follows:

1 $$\:Y\left(x\right)=\left(1-\frac{AET\left(x\right)}{P\left(x\right)}\right)\times\:P\left(x\right)$$

Where *AET*(*x*) is defined as the annual evapotranspiration occurring at pixel *x*, and *P*(*x*) is considered the annual precipitation occurring at pixel *x*. The evapotranspiration component of the water balance, *AET*(*x*)/*P*(*x*) for vegetated land categories, is based on Eq. (2) of the Budyko curve, which states that:

2 $$\:\frac{AET\left(x\right)}{P\left(x\right)}=1+\frac{PET\left(x\right)}{P\left(x\right)}-\:{\left[\:1+\:{\left(\frac{PET\left(x\right)}{P\left(x\right)}\right)}^{\omega\:}\right]}^{\frac{1}{\omega\:}}$$

Where *PET*(*x*) is defined as the potential evapotranspiration, and *ω*(*x*) is a non-physical parameter that depends on the local climate and soil properties. Potential evapotranspiration is further calculated using Eq. (3):

3 $$\:PET\left(x\right)=\:{K}_{c}\left({l}_{x}\right)\times\:E{T}_{0}\left(x\right)$$

Where *ET*_*0*_(*x*) is referred to as the reference evapotranspiration occurring at pixel *x*, and *K*_*c*_(*l*_*x*_) is the evapotranspiration coefficient of the land cover *l*_x_ on pixel *x*. *ET*_*0*_(*x*) represents local climatic conditions by analysing the evapotranspiration of the local vegetation type grown at that location.

### Model inputs

**Fig. 2:**
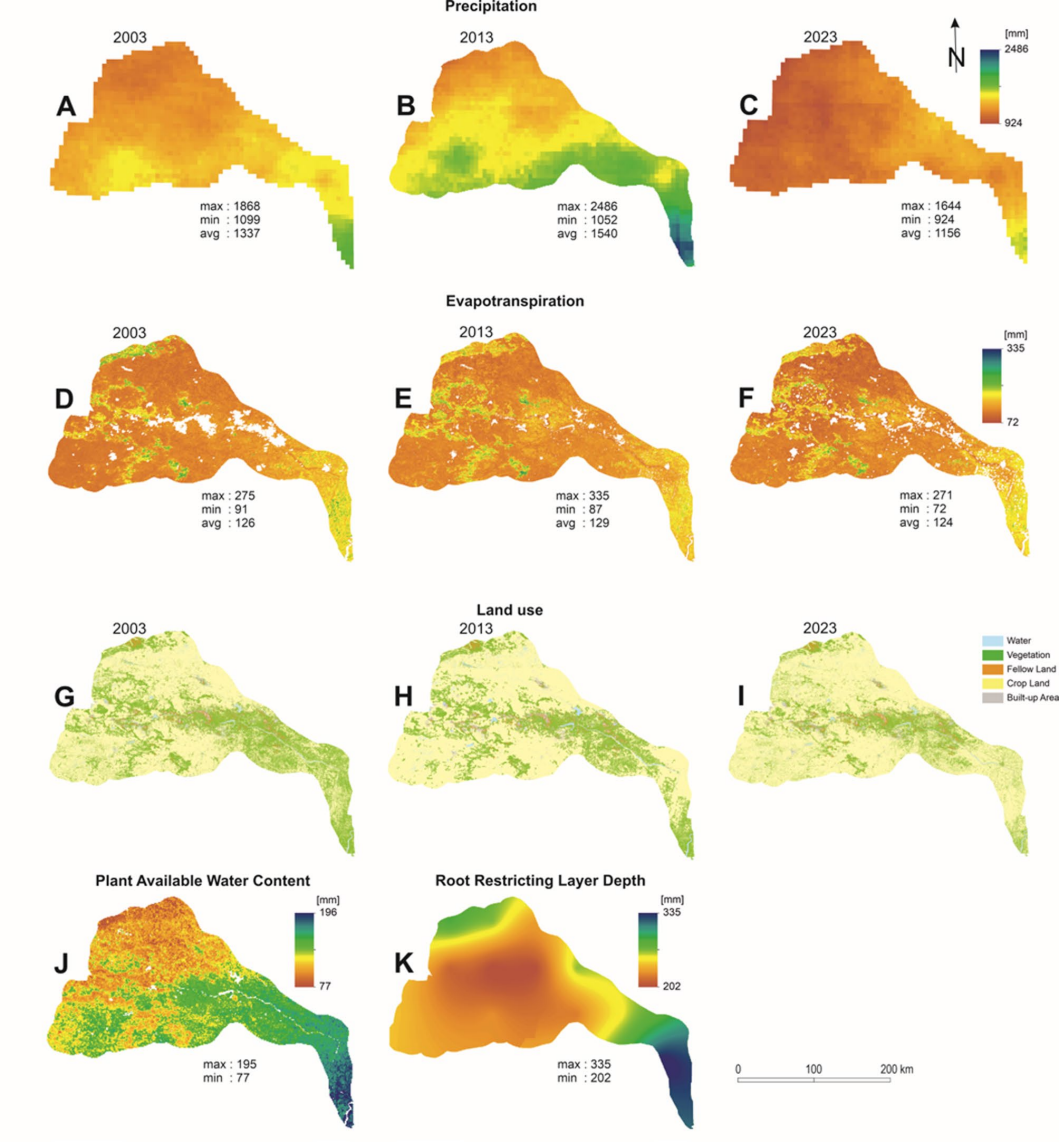
Inputs for the InVEST model, where (**A**), (**B**), and (**C**) are precipitation for 2003, 2013 and 2023, respectively; (**D**), (**E**), and (**F**) are evapotranspiration for 2003, 2013 and 2023, respectively; (**G**), (**H**) and (**I**) are land use for 2003, 2013 and 2023, respectively; (**J**) is the plant available water content in mm and (K) is the root-restricting layer depth. Source: authors’ own work.

The precipitation (Fig. 2A, B, and C) and evapotranspiration (Fig. 2D, E and F) rasters were created in the GEE platform using, respectively, CHIRPS and MODIS data. Root-restricting layer depth (Fig. 2K) and plant available water content (Fig. 2J) were created by clipping from global datasets (source mentioned in Table 1). Root-restricting layer depth is the depth to bedrock or the depth below ground where root penetration is restricted. It dictates water use by plants and can vary depending on biomes^35^. Coarse-grained soils at the surface are characterised by low water retention and thus facilitate deep infiltration, promoting the development of deep roots^36^. Rooting depth might vary due to differences in the local soil water profile caused by infiltration and drainage. Plant-available water content is a proportion derived from various common soil maps. It is the difference between the permanent wilting point and the volumetric field capacity fraction^30^. These two variables, root-restricting layer depth and plant-available water content, change only if the whole biome is changed, which is not the case in DRR. The raster for plant available water content was converted into fractions as per the recommendation of the user guide^40^. The data resolution of Table 1 was 30 m. As all the inputs need to be in the same resolution for model simulation, it was recommended in the user guide to resample all raster datasets into a common resolution. The model calculates the quantity of water that runs off each pixel from precipitation and evapotranspiration. This pixel-scale approach for computations enables us to depict the diversity of important water yield-influencing elements such as plant type, precipitation and soil type. However, the outputs in the per_pixel folder may be only helpful for intermediate computations.

Land use maps were prepared from the Landsat TM (for 2003) and OLI (for both 2013 and 2023) data. Land use estimation involved texture analysis, which was performed using the Gray Level Co-occurrence Matrix (GLCM) with additional indices like built-up index, normalised difference vegetation index (NDVI) and normalised difference water index (NDWI). Textural information, along with these indices, was used as input in the Random Forest (RF) algorithm. The output land use map had five classes – namely, water, vegetation, fallow land, cropland and built-up areas. All the inputs are depicted in Fig. 2.

Other inputs, like biophysical tables for 2003, 2013 and 2023, were prepared according to the user guide. Here, kc is a unitless number which refers to the evapotranspiration coefficient for each type of land use. The kc values for water, vegetation, fallow land, cropland and built-up areas were assigned as 1, 0.95, 0.5, 0.9 and 0.7, respectively. Researchers have used a range of kc values for the annual water yield model; however, we have used similar values of kc for water yield estimation of the upper Ganga basin^37^. The biophysical table constitutes different parameters for each land use class. Here, “lucode” is an integer and is different for each land use class, “lulc_veg” represents whether the land use class is vegetated or not, which helps in calculating actual evapotranspiration. A value of 1 in “lulc_veg” indicates the presence of vegetation in this class, whereas 0 values are used for waterbodies and urban areas.

**Fig. 3:**
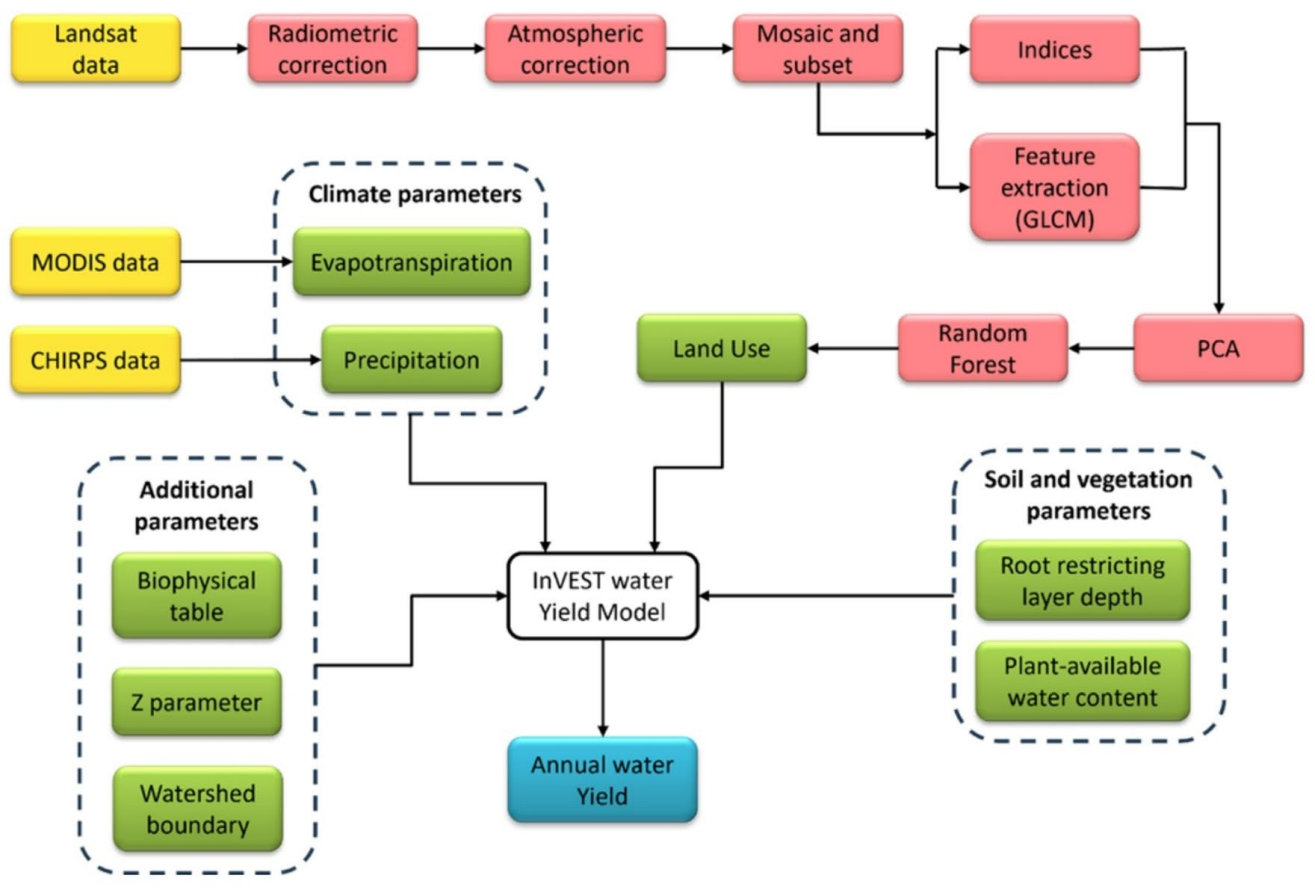
Flowchart of the methodology adopted in the present research, where yellow boxes indicate data sources, red boxes indicate intermediate steps, green boxes indicate inputs for the model, and blue box indicates output.

The Z parameter is the ecohydrological constant, which is an empirical measure that encapsulates the basin’s topography, rainfall intensity and seasonality of climate. It is an integer ranging from 1 to 30 and is also used as a calibration constant in model simulation. There are three available methods for the estimation of the Z parameter^38^ – namely (i) using streamflow data, which is unavailable in the present study area, (ii) incorporating globally available ω values, and (iii) using the number of precipitation days in a year. The third approach was used for the estimation of the Z parameter in this research. The overall methodological workflow is depicted in Fig. 3.

## Results

### Annual water yield of the DRR

**Fig. 4:**
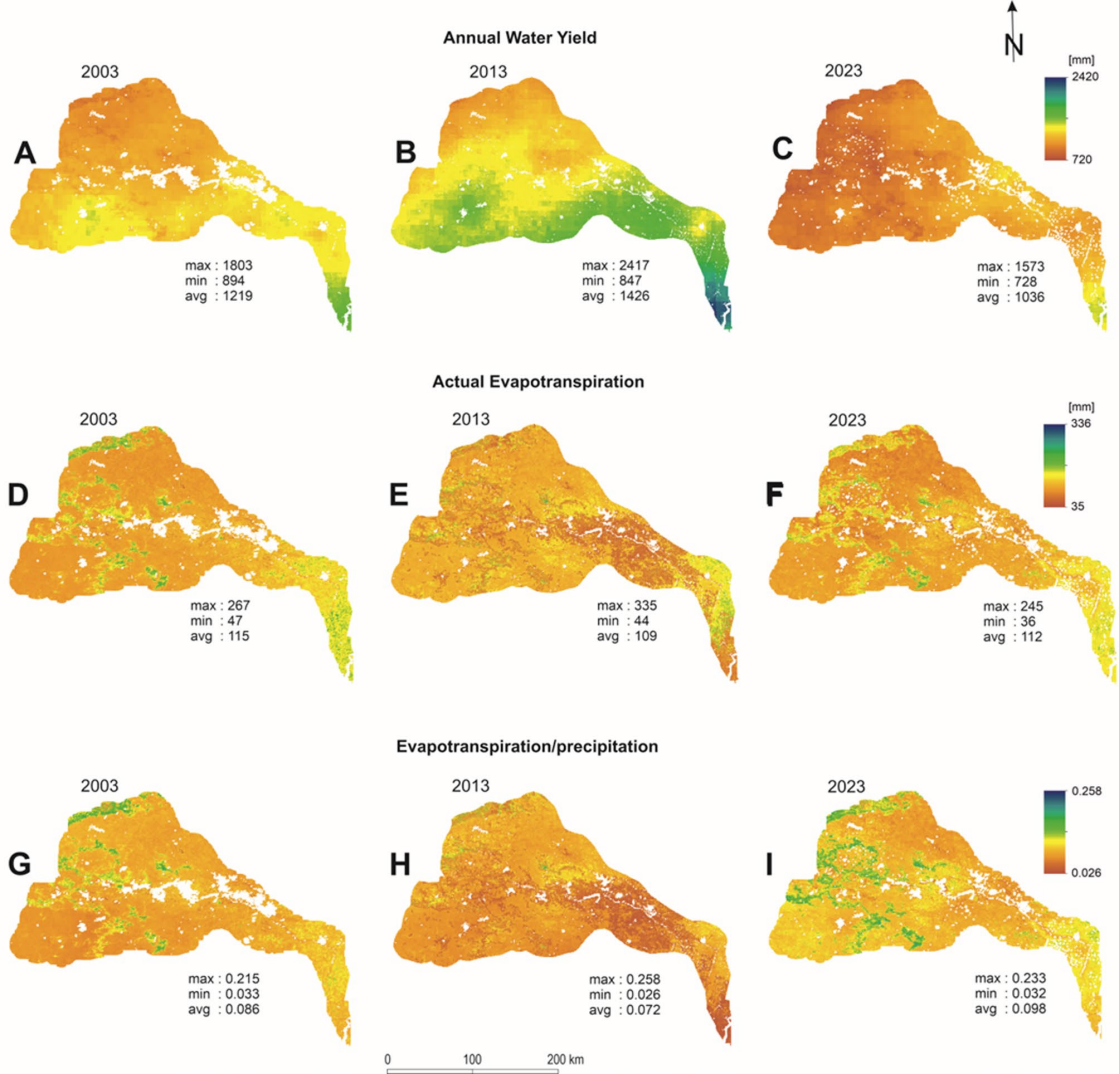
Outputs of the Annual water yield model, where: (**A**), (**B**) and (**C**) are annual water yield for 2003, 2013 and 2023, respectively; (**D**), (**E**) and (**F**) are Actual evapotranspiration for 2003, 2013 and 2023, respectively; and (**G**), (**H**) and (**I**) are actual evapotranspiration/precipitation for 2003, 2013 and 2023, respectively. Source: authors’ own work.

The outputs of the InVEST annual water yield model simulation are presented in Fig. 4. The white pixels indicate the presence of waterbodies/cloud cover in the original raster datasets. Figures 4 (A), (B) and (C) represent per-pixel annual water yield of the DRR for 2003, 2013 and 2023, respectively. For 2003, the lowest value obtained was 894 mm, whereas the highest value was 1,803 mm, with an average of 1,219 mm. In 2013, the lowest, highest and average values for water yield were 847 mm, 2417 mm and 1426 mm, respectively. In 2023, the lowest value for water yield per pixel obtained was 728 mm^,^ and the highest value was 1,572 mm^,^ with an average value of 1,036 mm. Therefore, the results suggest that the water yield of the DRR has been changing throughout the timescale. For 2003, 2013 and 2023 alike, lower water yield was observed along the western and north-western part of the study area, and higher values were observed near the southern part and the confluence of the Damodar River. Figures 4 (D), (E) and (F) represent the actual evapotranspiration, and (G), (H) and (I) represent the fraction of evapotranspiration and precipitation per pixel in the DRR for 2003, 2013 and 2023, respectively. The spatial distribution of the fraction suggests that the fraction was higher in 2023 compared to 2003 and 2013, which indicates that either evapotranspiration rose or precipitation dropped. The observation was further confirmed by analysing the evapotranspiration and precipitation input datasets (Fig. 2), which revealed that the average evapotranspiration was 126 mm for 2003, 129 mm for 2013, and 124 mm for 2023 (Fig. 2D, E and F), indicating very little change over two decades. On the other hand, the precipitation data reveal that the average values were 1,336 mm in 2003, 1540 mm in 2013 and 1,156 mm in 2023 (Figs. 2A and C). Although the average values of actual evapotranspiration did not change significantly between 2003 and 2023, as shown in Figs. 4 (D), (E) and (F), the precipitation might be the key factor controlling the fractions in Figs. 4 (G), (H) and (I). The total water yield for the whole DRR was 140,925 million m^3^ in 2003, 164,796 million m^3^ in 2013, and 119,840 million m^3^ for 2023. This indicates that, compared to 2003, water yield was lower in 2023 and higher in 2013.

### Contribution to the water yield for different land use classes

Supplementary Table 1 represents the water yield for each land use class in the DRR. The land use map was generated from Landsat images, where the overall accuracies obtained for 2003, 2013, and 2023 were 89.45%, 87.45% and 91.8%, respectively. (Accuracy information is provided in Supplementary Tables 2, 3 and 4.) However, the overall kappa values for 2003, 2013 and 2023 were similar to one another, at 0.86, 0.88 and 0.87, respectively. The water yield was highest for fallow land in the DRR in 2003, 2013 and 2023, with average values of 1,205 mm, 1511 mm and 1,022 mm, respectively. It was followed by built-up areas (average value of 1,198 mm for 2003, 1,423 mm for 2013 and 1,008 mm for 2023), cropland (average value of 1,188 mm in 2003, 1,396 for 2013, and 978 mm for 2023), and vegetation (average value of 1,181 mm for 2003, 1,364 mm for 2013, and 945 mm for 2023).

### Potential reasons for reduced water yield in the DRR

**Fig. 5:**
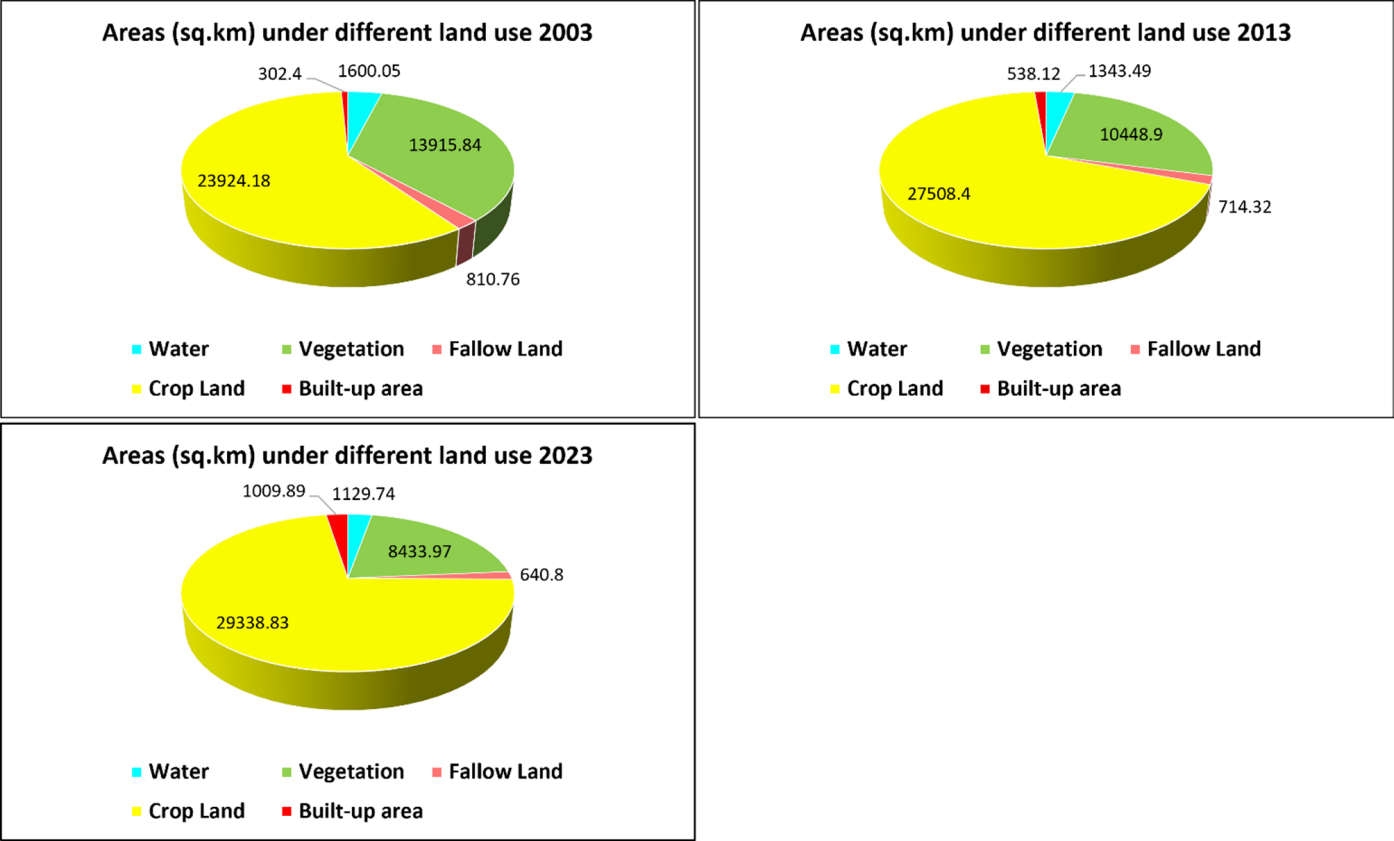
Areas under different land use for 2003, 2013, and 2023.

The results presented in the previous section suggest that water yield declined in the DRR from 2003 to 2023, with a higher yield reported in 2013. While reduced precipitation is somewhat responsible for the decreased water yield, further investigation has been carried out to observe the effects of land use on this phenomenon. Figure 5 depicts the areas under different land use classes in the DRR for 2003, 2013 and 2023. It is easily observable that built-up areas increased from 302.4 km^2^ in 2003 to 538.12 km^2^ in 2013, to 1,009.89 km^2^ in 2023, indicating rapid growth. On the other hand, vegetation cover dropped from 13,915.84 km^2^ in 2003 (34.31% of the total area) to 10448.9 km^2^ in 2013 (25.76% of the total area) to 8,433.97 km^2^ in 2023(20.79% of the total area). Similarly, areas under waterbodies and fallow land were also reduced, and cropland increased by 5,414 km^2^ between 2003 and 2023 (59% of the total area in 2003 to 67.83% of the total area in 2013 to 72.34% of the total area in 2023) to accommodate the growing population and increased food demand. Increased impervious surfaces due to urbanisation and reduced vegetation cover, along with local waterbodies, are thought to be the main reasons behind reduced water yield in the DRR.

### Sensitivity analysis

A sensitivity analysis was carried out to understand the dependency of the water yield on selected parameters. Tables 2 and 3 represent the sensitivity analysis results for precipitation and evapotranspiration, respectively. The tables show that the InVEST annual water yield model is more sensitive to precipitation than to evapotranspiration, which was also found by^38,39^. In this research, an increase of 5% in precipitation resulted in a 5.48% increase in water yield, whereas a 10% increase in precipitation resulted in a 10.97% increase in water yield. In the case of evapotranspiration, a 5% increase resulted in a 0.48% decrease in water yield and vice versa, which proves that the model is more sensitive to precipitation than to evapotranspiration. Note that the water yield varies linearly with changes in precipitation or evapotranspiration, which might be due to the model characteristics (the model does not take human consumption into account), which was also found by other studies^40^. The model was also simulated with different climate and land use datasets to observe the changes in water yield with respect to land use (Table 4). The average annual water yield results obtained with the climate data of 2003 and the land use of 2013 and 2023 were 1,226 mm and 1,220 mm, respectively. The average annual water yield result obtained with both land use and climate data of 2003 was 1,219, indicating very little or no change. Similarly, the average annual water yield results obtained with the climate data of 2023 and the land use of 2003 and 2013 were 1,035 mm and 1,043 mm, respectively. The average annual water yield result obtained with both land use and climate data of 2023 was 1,036 mm, revealing virtually no change.

Sensitivity analysis was also carried out for the Z parameter, which resulted in almost identical water yields for Z values 5 to 25. This might be because higher Z values are not sensitive to water yield, and this has been proven particularly for humid zones where precipitation is high^31,41^. Several researchers have found the model sensitive to Z values^42,43^, but it is only applicable if the Z values are low, i.e., the region is predominantly arid. The DRR basin falls under a tropical climate and experiences more than 100 rain events per year due to the south-west monsoon; thus, Z values remain on the higher side and do not influence the annual water yield.

**Table 2:** Sensitivity of the annual water yield model to precipitation.

PPT variation	Water yield (in M m^3^)	Change in water yield
+ 5%	144,541.46	5.48% increase
+ 10%	152,059.13	10.97% increase
−5%	129,506.14	−5.48% decrease
−10%	121,988.51	−10.97% decrease

**Table 3:** Sensitivity of the annual water yield model to evapotranspiration.

ET variation	Water yield (M m^3^)	Change in water yield
+ 5%	136,357.33	−0.48% decrease
+ 10%	135,690.88	−0.97% decrease
−5%	137,690.27	0.48% increase
−10%	138,356.75	0.97% increase

**Table 4:** Sensitivity of the annual water yield model to different land use scenarios.

Scenarios	Maximum water yield (mm)	Minimum water yield (mm)	Mean water yield (mm)
Climate data of 2003 with land use of 2013	1,804	884	1,226
Climate data of 2003 with land use of 2023	1,803	894	1,220
Climate data of 2023 with land use of 2003	1,572	728	1,035
Climate data of 2023 with land use of 2013	1,583	718	1,043

## Discussion

In the era of climate change, the estimation of water yield is crucial for both ecosystem services and water management purposes. This research evaluates the annual water yield of the DRR using the InVEST annual water yield model and geospatial techniques. The InVEST annual water yield model has been extensively used worldwide for water yield estimation in watershed and sub-watershed scales. This model has the capability to successfully simulate the percentage of baseflow to total flow^44^, show the spatial distribution of baseflow^45^, simulate water yield in future land use scenarios^46^, etc. The model can assist water resource managers in planning irrigation schedules, assessing the effects of climate and land use changes, and determining optimal scenarios. However, this model has several limitations, such as not taking intra or inter-annual variability of water supply into account^42^. Factors linked to ecosystem services, like flooding and irrigation, depend on water yield, which is not represented by the model. The model also disregards groundwater and lateral flows, thereby neglecting intricate land use patterns or underlying geology^30^. The model also handles consumptive water usage in a very basic manner, which helps in reducing overestimation and enhancing model performance.

**Fig. 6:**
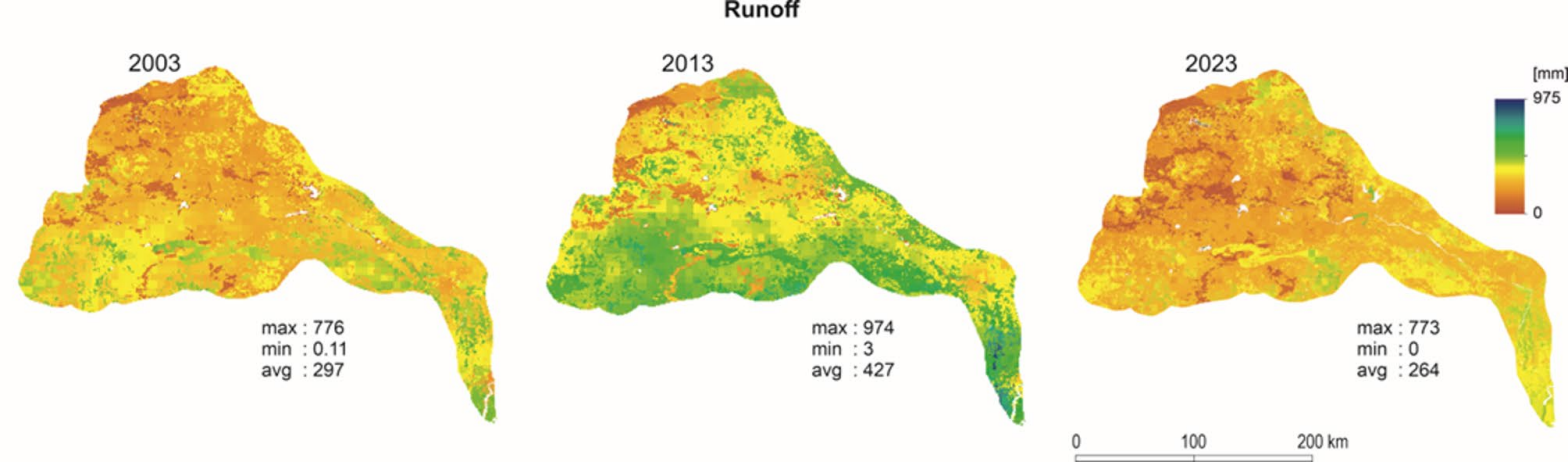
Runoff output (per pixel) from the SCS CN model. Source: authors’ own work.

The results obtained in this study imply a reduced water yield from 2003 to 2023 in the DRR, with an increase in the year 2013. To confirm the observation and check the pattern of the runoff in this period, a rainfall-runoff model was also simulated in the GEE platform. As observed discharge data were not accessible, validation was carried out indirectly using rainfall-runoff simulations to assess the reliability of the InVEST model outputs under data-scarce conditions. The basic code for the model was developed by Jain et al.^47^ and the functionality is dependent on the Soil Conservation Services curve number (SCS CN). The output of the model is presented in Fig. 6. The mean runoff value was 297 mm for 2003, 427 mm for 2013, and 264 mm for 2023. The rainfall-runoff model also supports the observation that water yield, as well as runoff, is abruptly changing in the DRR.

Although there is evidence in the literature that runoff, as well as the carrying capacity of the Damodar River, decreased after the construction of the dam and reservoir^48^, the results obtained from this study nevertheless do not reflect a decreasing trend. The decreased carrying capacity could be the main reason behind the frequent floods in the lower catchment, but it does not dictate water yield or runoff. Further investigation on the matter was carried out to identify the main regulating factor of the water yield in the InVEST model simulation. The model theoretically might be sensitive to land use, precipitation and evapotranspiration. The results obtained from Table 4 suggest that this water yield model is not sensitive to land use changes, as previously thought. There was an observable change in the land use from 2003 to 2013, where built-up areas and cropland increased, and vegetation cover decreased. This should result in decreased water yield, but the annual water yield increased in 2013 compared to 2003. Hence, it was proven that land use has very little impact on the InVEST annual water yield model output. The existing literature suggests that a short-term analysis does not always reflect the relationship between land use and water yield. For example, a 25-year study of the effect of land use on the water yield reveals very little change^49^, whereas long-term observation of land use changes revealed a significant change in water yield^50^. The studies that have observed changes in water yield have considered longer time periods, such as 1955–2005^51^, or 1935–2005^52^ as it was already proved that there might be a considerable delay an observable change in water yield due to changes in land use^53^.

**Fig. 7:**
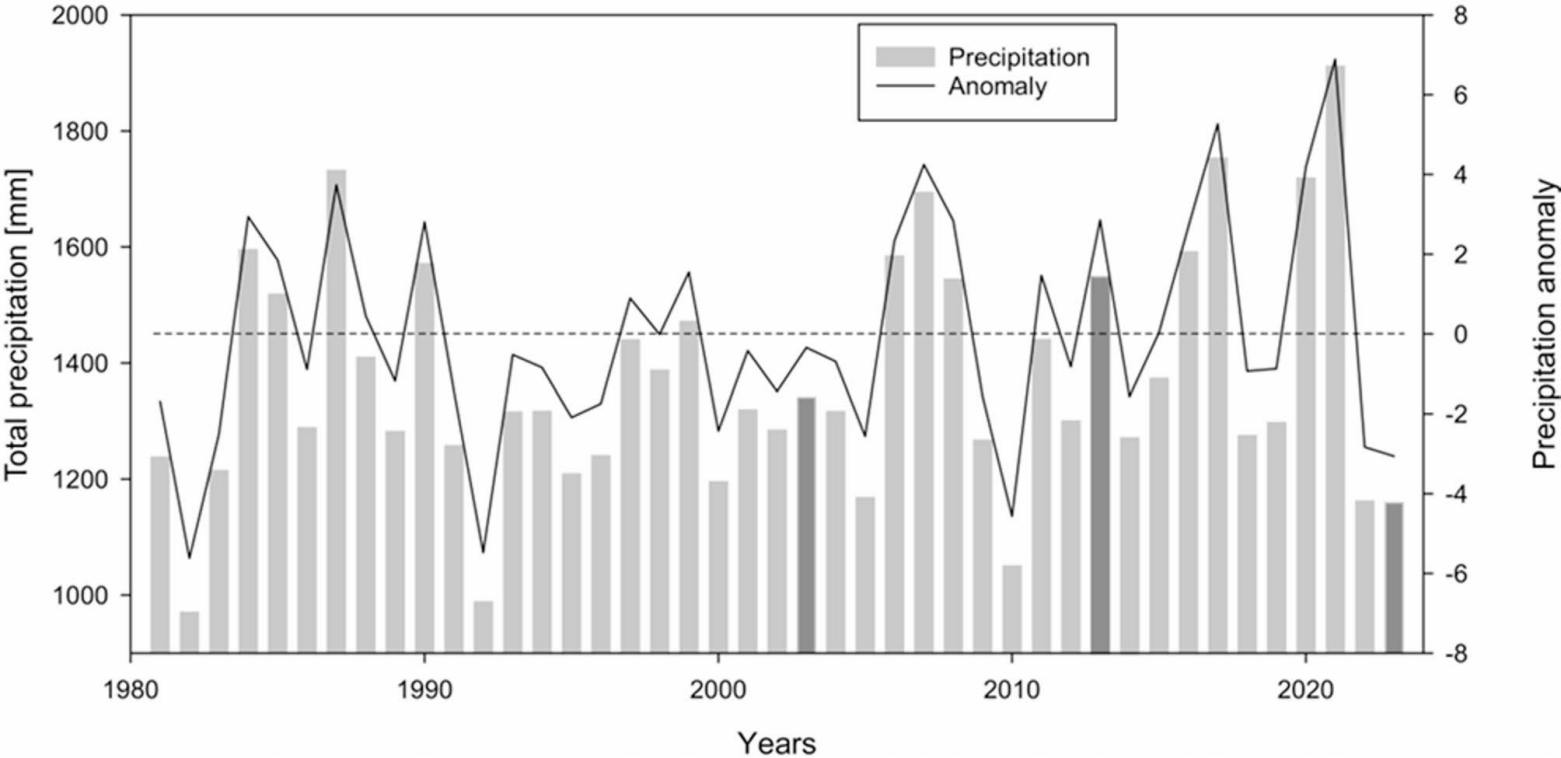
Annual precipitation and precipitation anomaly over the DRR.

The two factors left were evapotranspiration and precipitation, and it was already established in the sensitivity analysis that precipitation is significantly more sensitive to annual water yield than evapotranspiration. The primary element of the terrestrial hydrological cycle is precipitation^54^, and variations in precipitation have a greater influence on the hydrology of a rain-fed river basin like the DRR. The study area lies in two eastern states of India – Jharkhand and West Bengal. Available literature suggests that between 1901 and 2011, a negative trend in precipitation was observed in Jharkhand^55^. In West Bengal, apart from the northern Himalayan and southern coastal regions, a negative trend of precipitation was also observed^56^. Not only precipitation but also the average and maximum temperatures have increased in both Jharkhand and West Bengal^57^, as observed from long-term climatological data^58^. Therefore, the question emerges as to why the long-term decreasing trend of precipitation does not follow the results from the InVEST water yield model simulation. It was found by several authors in the literature that the InVEST model is essentially a scenario-based simulation, so it can capture temporal snapshots, rather than a continuous trend. To confirm the observation, total annual precipitation with precipitation anomaly was plotted (Fig. 7) using the CHIRPS precipitation data, which was also used for the model simulation. The model-simulated years are made darker to emphasise the fact that precipitation regulates the annual water yield model output. The water yield was found to be highest in 2013, followed by 2003, and the lowest in 2023. The total precipitation in those years corroborates the fact.

Considering that the foundation of the InVEST water yield model is built on the Budyko curve and average annual precipitation, it makes sense that the results will be more sensitive to precipitation data. The Budyko curve illustrates the correlation between potential evapotranspiration/precipitation (PET/P) and actual evapotranspiration/precipitation (AET/P). The AET/P or evaporative index (EI) is plotted against the PET/P or dryness index (DI) to observe the characteristics of the basin. Budyko identified two types of catchments, where evapotranspiration (ET) is restricted by either energy or water supply. Climate change affects evapotranspiration by influencing net radiation and vapour pressure deficit, and the availability of water in the catchment intercepted by the canopy or stored on the ground surface or in the soil. A catchment with DI < 1 is considered to be humid and energy-limited, whereas one with DI > 1 is considered to be dry and water-limited. For the DRR, the EI is presented in Fig. 4 (G, H and I). From the model outputs, the average PET values for 2003, 2013 and 2023 were found to be 115 mm, 109 mm and 112 mm, respectively. Thus, both the EI and DI values are very low for the DRR, making it an energy-limited basin, not a water-limited basin. In an energy-limited basin, the primary regulating factor of evapotranspiration is solar radiation and temperature. Therefore, unless there is a clear change in the climatic variables that control evapotranspiration, the annual water yield will be controlled mainly by precipitation. The Budyko curve could also offer a helpful framework for predicting how catchments would react to shifting weather patterns. The capacity of a catchment to withstand the effects of climate change while preserving the hydrological function is known as “hydrological resilience”^59^. Hydrologically resilient catchments should react to changing conditions and maintain their DI and EI points close to the Budyko curve, rather than being set at a certain spot on the diagram.

### Watershed management in the DRR: opportunities and challenges

Hydrological alterations disrupt the natural flow patterns of almost 60% of the world’s rivers^60^. Protecting aquatic resources from the influence of dams and water extraction in rivers is a complex topic in sustainable river basin management. The magnitude, timing, frequency and rate of changes in water resources are crucial to the long-term sustainability and proper utilisation of such resources. Management of water resources includes maintaining healthy river ecosystems and other features, including fish and wildlife habitat, and water quality^61^. The construction of the dam and reservoir has altered the hydrology and morphology of the Damodar River^62^. As a result, the river has experienced heavy sedimentation, and the lower part of the channel is no longer capable of storing floodwater inside its valley during peak monsoon seasons^48^. For this reason, every year during the late monsoon, when the DVC releases excess water, the low-lying riparian areas of the lower Damodar Basin typically experience low-magnitude flooding that destroys habitats and crops. With just four dams and Tenughat reservoirs, the projected project’s maximum storage capacity is only 3,591 million m^3^, or 55% of the storage capacity that was initially planned. Maithon and Panchet, the final two terminal dams are situated near a topographical slope break; as a result, the reservoirs receive a lot of sediment load from the Damodar and Barakar tributaries (Supplementary Table 5), and one of the main issues that is affecting the downstream flood control systems is the siltation of the reservoirs^63^.

**Fig. 8:**
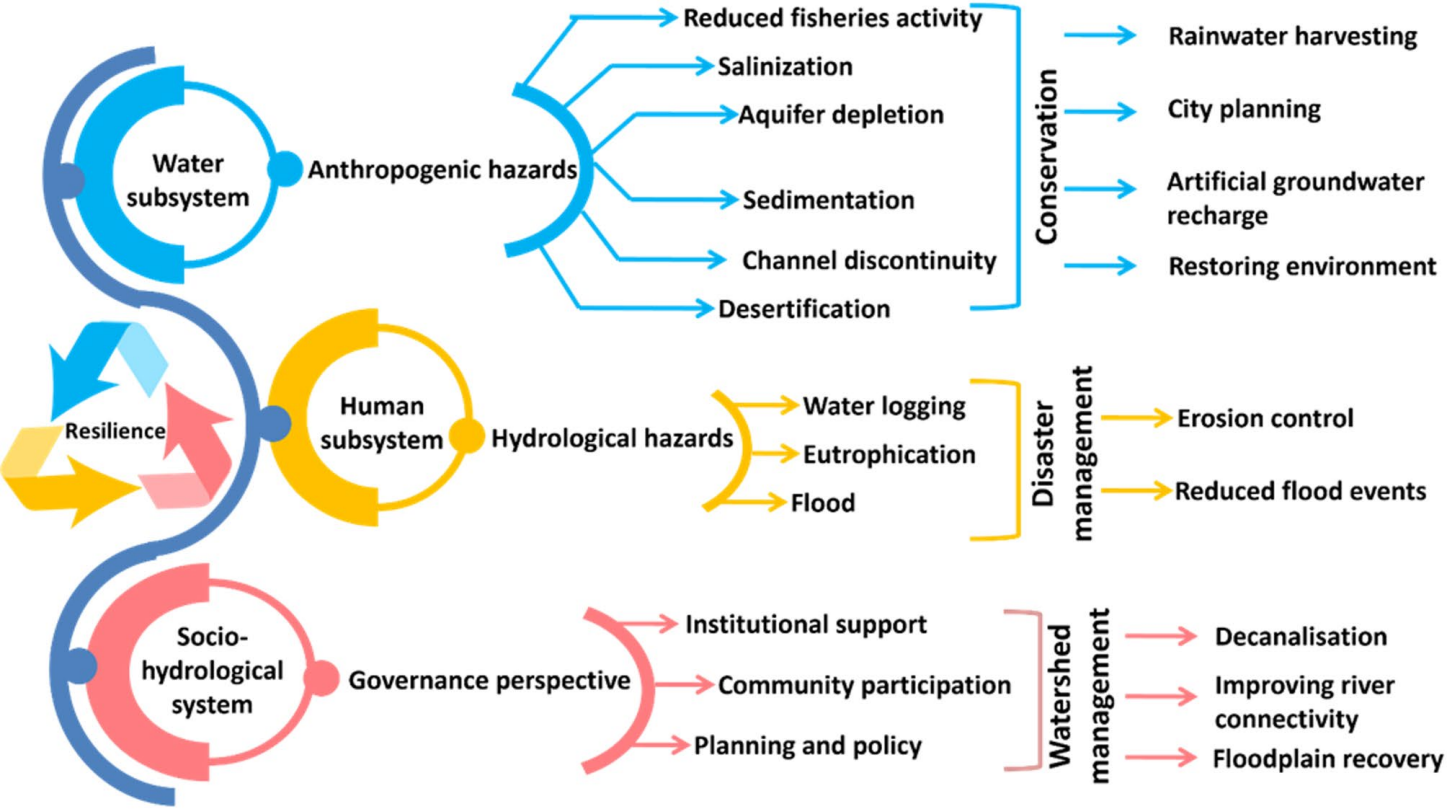
Pathways for building hydrological resilience in the DRR.

Therefore, this article also suggests some steps to build hydrological resilience in the DRR. In the domains of academia and public policy, resilience is becoming more and more important in the coupled human–water context. Examples of these include the conservation of aquatic ecosystems, hydrological risk management and sustainable water use and development^64^. This framework is based on managing the three sub-systems (Fig. 8) that represent human–water couplings: (a) the water subsystem, which indicates hydrological resilience to anthropogenic hazards; (b) the human subsystem, which represents social resilience to hydrological hazards; and (c) the socio-hydrological system, which identifies that humans and water are interdependent.

These three types of human–water couplings each have their own resilience framing. These three categories highlight the underlying connections between people and water while offering several angles from which to view and comprehend socio-hydrological systems and human–water interactions. The anthropogenic hazards in the water sub-system include reduced fishing activity, salinisation, aquifer depletion, sedimentation, channel discontinuity and desertification. These phenomena necessarily decrease the resilience of a river basin, which is directly influenced by human land-use change. Conservation approaches like rainwater harvesting, city planning to decrease impervious surfaces, artificial groundwater recharge and forestation could potentially solve the issues and strengthen resilience. The human subsystem represents hydrological hazards like flooding, waterlogging and eutrophication. Disaster management techniques might reduce the recurring flood events in DRR, and eutrophication could be reduced by decreasing nutrient load and ratio, applying algaecides and herbicides, etc. Although five dams were built to reduce frequent flooding in the lower DRR, monsoon flooding is still a persistent issue. The socio-hydrological subsystem consists of governance initiatives like planning, policies, community participation and institutional support. Proper watershed management strategies could help in building socio-hydrological resilience in DRR; these include decanalisation, improving river connectivity and floodplain recovery. For agricultural purposes, several canals have been built to supply fresh water to the adjacent areas. On the other hand, many natural water channels have also been cut off for development activities. These activities have harmful effects on the resilience of the DRR as agricultural canals bring substantial amounts of nutrients that induce eutrophication, and the discontinued natural canals reduce runoff. Hence, building holistic watershed management in DRR is a complex problem and needs a multidimensional approach.

## Conclusions

This study deals with the simulation of the InVEST annual water yield model for the DRB by using geospatial datasets and the RF machine learning algorithm. The results obtained from the model simulations indicate an increase in water yield from 2003 to 2013, followed by a decrease in water yield in 2023. The increasing and decreasing water yield is also corroborated by the rainfall runoff model simulations for 2003, 2013 and 2023. To explain the pattern of water yield in the DRB, a series of analyses was carried out to search for possible reasons behind such observations. The land use analysis indicates a continuous decrease in vegetation cover and a corresponding increase in cropland and built-up areas between 2003 and 2023. The sensitivity analysis revealed that water yield is profoundly influenced by precipitation rather than evapotranspiration. An increase of precipitation by 10% resulted in an increase in the water yield by almost 11%, whereas an increase in evapotranspiration resulted in only about 1% decrease in water yield. The influence of land use on the water yield was estimated using a set of scenario-based analyses, which indicate that land use has very little sensitivity to water yield in the DRB. Therefore, precipitation is clearly the predominant controlling factor for water yield in the DRB, which is consistent with the fact that the Damodar River is essentially non-perennial and the main source of water is the monsoonal rainfall. The intense monsoon rain results in floods despite the construction of reservoirs in the upper catchment. A watershed management framework has been proposed in this research, based on the three main conceptual subsystems. Future research should focus on adaptation and mitigation strategies discussed in the framework to create hydrological resilience in the DRB.

## Supplementary Information

Below is the link to the electronic supplementary material.

Supplementary Material 1

## Data Availability

The datasets generated during the current study are available from the corresponding author upon reasonable request.
