# Supplementary material for: A modelling approach to assess the impacts of climate dynamics and anthropogenic pressure on water yield in the Damodar River basin
Source: Sci Rep. 2025 Nov 23;15:45181. doi: 10.1038/s41598-025-29098-9 (PMC12749323; doi:10.1038/s41598-025-29098-9)
Supplement: Supplementary file 1 — Supplementary Material 1 [file 41598_2025_29098_MOESM1_ESM.docx]

|  | 2003 | | | 2013 | | | 2023 | | |
| --- | --- | --- | --- | --- | --- | --- | --- | --- | --- |
| Land use | Min | Max | Mean | Min | Max | Mean | Min | Max | Mean |
| Vegetation | 893.68 | 1,463.81 | 1,181.40 | 847.64 | 2,354.39 | 1,364.61 | 625.58 | 1,221.11 | 945.12 |
| Fallow land | 1,003.41 | 1,510.97 | 1,205.79 | 943.19 | 2,416.66 | 1,511.50 | 696.72 | 1,266.20 | 1,022.19 |
| Cropland | 905.65 | 1,471.34 | 1,188.08 | 868.64 | 2,360.12 | 1,396.10 | 633.84 | 1,226.12 | 978.12 |
| Built-up | 951.03 | 1,490.31 | 1,198.17 | 913.97 | 2,311.76 | 1,423.32 | 666.89 | 1,246.16 | 1,008.72 |

**Supplementary:**

**Supplementary Table 1.** Water yield (in m3/per pixel) for each land use class

| 2003 | | | | | | | | | | | | |
| --- | --- | --- | --- | --- | --- | --- | --- | --- | --- | --- | --- | --- |
|  | Water | Vegetation | Fallow Land | Crop Land | Settlement | Total | PA (%) | UA (%) | OCA (%) | Kappa Statistics | Overall Kappa |  |
| Water | 4 | 0 | 0 | 0 | 0 | 4 | 80 | 100 | 89.45 | 1 | 0.86 |  |
| Vegetation | 0 | 32 | 1 | 0 | 3 | 36 | 94.12 | 88.89 |  | 0.87 |  |  |
| Fallow Land | 0 | 2 | 61 | 2 | 5 | 70 | 83.56 | 87.14 |  | 0.82 |  |  |
| Crop Land | 1 | 0 | 7 | 74 | 2 | 84 | 97.37 | 88.1 |  | 0.83 |  |  |
| Settlement | 0 | 0 | 4 | 0 | 58 | 62 | 85.29 | 93.55 |  | 0.91 |  |  |
| TOTAL | 5 | 34 | 73 | 76 | 68 |  |  |  |  |  |  |  |

**Supplementary Table 2.** Accuracy table for land use classification of 2003

| 2013 | | | | | | | | | | | |
| --- | --- | --- | --- | --- | --- | --- | --- | --- | --- | --- | --- |
|  | Water | Vegetation | Fallow Land | Crop Land | Settlement | TOTAL | PA (%) | UA (%) | OCA (%) | Kappa Statistics | Overall Kappa |
| Water | 8 | 0 | 0 | 0 | 0 | \| 8 \| \| --- \| | 100 | 100 | 87.45 | 1 | 0.88 |
| Vegetation | 0 | 25 | 4 | 0 | 2 | 31 | 86.21 | 80.65 |  | 0.86 |  |
| Fallow Land | 0 | 3 | 68 | 6 | 2 | 79 | 86.08 | 86.08 |  | 0.83 |  |
| Crop Land | 0 | 0 | 5 | 131 | 0 | 136 | 93.57 | 93.57 |  | 0.89 |  |
| Settlement | 0 | 1 | 2 | 3 | 19 | 25 | 82.61 | 76 |  | 0.81 |  |
| TOTAL | 8 | 29 | 79 | 140 | 23 |  |  |  |  |  |  |

**Supplementary Table 3.** Accuracy table for land use classification of 2013

| 2023 | | | | | | | | | | | |
| --- | --- | --- | --- | --- | --- | --- | --- | --- | --- | --- | --- |
|  | Water | Vegetation | Fallow Land | Crop Land | Settlement | TOTAL | PA (%) | UA (%) | OCA (%) | Kappa Statistics | Overall Kappa |
| Water | 5 | 0 | 0 | 0 | 0 | 5 | 100 | 100 | 91.8 | 1 | 0.87 |
| Vegetation | 0 | 21 | 2 | 0 | 0 | 23 | 84 | 91.3 |  | 0.9 |  |
| Fallow Land | 0 | 3 | 75 | 0 | 5 | 83 | 87.21 | 90.36 |  | 0.85 |  |
| Crop Land | 0 | 0 | 7 | 119 | 1 | 127 | 100 | 93.7 |  | 0.88 |  |
| Settlement | 0 | 1 | 2 | 0 | 15 | 18 | 71.43 | 83.33 |  | 0.82 |  |
| TOTAL | 5 | 25 | 86 | 119 | 21 |  |  |  |  |  |  |

**Supplementary Table 4.** Accuracy table for land use classification of 2023

| Reservoir name | Construction year | Gross Storage Capacity (MCM) | Live Storage Capacity (MCM) |
| --- | --- | --- | --- |
| Tilaiya | 1953 | 141 | 75 |
| Konar | 1955 | 337 | 276 |
| Maithon | 1957 | 1,093.54 | 441.64 |
| Panchet | 1959 | 1,497.5 | 1,327.17 |
| Tenughat | 1985 | 1,024.7 | 814.8 |

**Supplementary Table 5.** Details of the five reservoirs (construction year and storage capacity).
